# Enhancing low contrast color images via pythagorean interval-valued fuzzy sets and CLAHE

**DOI:** 10.1371/journal.pone.0354362

**Published:** 2026-08-03

**Authors:** Uma Maheswari S, Jagatheswari S

**Affiliations:** Department of Mathematics, School of Advanced Sciences, Vellore Institute of Technology, Vellore, Tamilnadu, India; Anhui University, CHINA

## Abstract

This study presents a pythagorean fuzzy set-based framework combined with contrast-limited adaptive histogram equalization (CLAHE) for low-light color image enhancement. Low-light color images often suffer from poor contrast, reduced visibility, and brightness distortion. To address these challenges, the proposed method employs pythagorean fuzzy sets to model pixel-level uncertainty more flexibly than conventional fuzzy representations. The method first transforms the input image into a pythagorean fuzzy image using a parameterized nonlinear membership mapping and then applies adaptive CLAHE to enhance contrast while preserving color fidelity. Experimental results on benchmark datasets demonstrate that the proposed approach improves visual quality and achieves competitive quantitative performance compared with existing enhancement techniques. Performance is evaluated using entropy, absolute mean brightness error, contrast improvement index, correlation coefficient, structural similarity index, and the natural image quality evaluator. The results highlight the effectiveness of integrating pythagorean fuzzy uncertainty modeling with adaptive contrast enhancement for low-light color image processing.

## 1. Introduction

Image enhancement plays a critical role in improving visual perception for computer vision, pattern recognition, and digital image processing applications. Images captured under low-light conditions often suffer from poor contrast, reduced visibility, brightness distortion, and loss of fine details, which degrade subsequent analysis and interpretation. Developing effective techniques to enhance low-light color images while achieving controlled brightness adjustment and preserving color fidelity therefore remains an active research problem.

Histogram-based contrast enhancement techniques have been widely explored due to their simplicity and effectiveness. Histogram Equalization (HE), introduced by Pizer et al. [1], laid the foundation for adaptive contrast enhancement. Numerous variants have been proposed to address the limitations of conventional HE. Kapoor et al. [2] improved color quality in underexposed images using HE, while Ibrahim et al. [3] proposed dynamic histogram equalization to preserve brightness. Contrast Limited Adaptive Histogram Equalization (CLAHE) has gained prominence for controlling over-enhancement and noise amplification in low-light images, with approaches based on rayleigh distributions, laplacian filtering, and adaptive gamma correction demonstrating improved visibility, color quality, and naturalness [4–6].

More recent studies have combined CLAHE with filtering, tone mapping, retinex-based correction, and optimization strategies to improve contrast and brightness consistency in challenging illumination conditions [7–13]. Despite these advances, histogram-based approaches primarily operate on intensity distributions and do not explicitly model uncertainty in low-light regions. As a result, they may produce over-enhancement, brightness shifts, or color distortion when applied to severely degraded images. To overcome these limitations, fuzzy set theory has been widely employed in image enhancement. Since the introduction of fuzzy sets by Zadeh [14], various fuzzy-based methods have been proposed, including type-2 fuzzy logic [15], intuitionistic fuzzy generators [16], and fuzzy histogram equalization techniques [17]. More recent works have explored adaptive fuzzy models, clustering-based enhancement, and optimization-driven fuzzy frameworks to improve robustness under uneven illumination [18–20]. Fuzzy learning-based and neighborhood-aware enhancement strategies have also been explored to improve local contrast and detail preservation under complex lighting conditions [21–23]. Recent approaches have also explored separating brightness and color components using learning-based strategies for low-light enhancement [24]. Pythagorean fuzzy sets (PFS), an extension of intuitionistic fuzzy sets, offer greater flexibility in uncertainty modeling by relaxing the constraint between membership and non-membership degrees. This property has motivated their application in medical image segmentation and fusion tasks [25,26]. However, their use in low-light color image enhancement remains relatively limited. Existing PFS and interval-valued intuitionistic fuzzy set-based enhancement methods often rely on fixed or heuristic membership generators and apply contrast enhancement techniques, such as CLAHE, as independent post-processing steps [27,28]. Other fuzzy enhancement operators and generators have been proposed to adaptively regulate pixel membership and local contrast, although their effectiveness may degrade for high-resolution or severely low-light images [29, 30]. Such sequential designs may not sufficiently adapt to diverse contrast distributions and can lead to brightness inconsistency or over-enhancement under highly nonuniform illumination.

Although several fuzzy and pythagorean fuzzy-based image enhancement methods have been reported, their design philosophies differ from the proposed approach. Intuitionistic fuzzy and interval-valued intuitionistic fuzzy methods typically employ predefined membership mappings and treat contrast enhancement as a separate processing stage, which limits their adaptability in severely low-light regions where pixel intensities are highly compressed and noise sensitivity is elevated. Existing pythagorean fuzzy-based studies primarily focus on segmentation, fusion, or decision-making applications, and their direct applicability to low-light color image enhancement is therefore limited. In contrast, the proposed method introduces a parameterized nonlinear membership mapping tailored for low-light contrast redistribution and integrates pythagorean fuzzy modeling with CLAHE within a unified enhancement framework. This joint formulation enables uncertainty modeling and local contrast enhancement to cooperatively regulate brightness, contrast, and color fidelity, rather than treating these aspects independently.

Motivated by these observations, this paper presents a hybrid low-light color image enhancement framework that combines pythagorean fuzzy modeling with contrast-limited adaptive histogram equalization. The proposed approach first constructs a pythagorean fuzzy image using a parameterized nonlinear membership function to represent pixel-level uncertainty in low-light regions. CLAHE is then applied to adaptively enhance local contrast while controlling over-enhancement and preserving color fidelity. By jointly leveraging uncertainty-aware fuzzy modeling and contrast-limited histogram equalization, the proposed framework aims to achieve balanced contrast enhancement and controlled brightness adjustment across a wide range of low-light color images.

The main contributions of this paper are summarized as follows:

- A parameterized nonlinear increasing membership function is introduced to construct pythagorean fuzzy images, enabling flexible and adaptive modeling of pixel-level uncertainty in low-light color images.
- A unified low-light image enhancement framework is proposed by integrating pythagorean fuzzy modeling with CLAHE, allowing uncertainty modeling and contrast enhancement to jointly regulate brightness, contrast, and color fidelity.

### 1.1. Organization

This manuscript is organized as follows. Section 2 describes the initial steps involved in constructing the fuzzy set. Section 3 presents the proposed pythagorean fuzzy image enhancement method. Section 4 reports the experimental results and provides a detailed analysis of the proposed approach. Section 5 presents an ablation study highlighting the contributions of individual components. Section 6 analyzes the computational time complexity of the proposed method in comparison with existing approaches. Section 7 discusses the advantages and limitations of the method. Finally, Section 8 concludes the paper and outlines potential directions for future research.

## 2. Preliminaries

### 2.1. Fuzzy set

Let X be the universe of discourse x ∈ X  then a fuzzy set A in X is defined as a set of ordered pairs as


$$\begin{array}{c} A=\{(x,\mu_{A}(x)):x\in X\} \end{array}$$ (1)

where $\mu_{A}:X\to [0,1]$ is called the membership function of x in A.

### 2.2. Intuitionistic fuzzy set [31,32]

An intuitionistic fuzzy set $A^{*}$ in X is defined as


$$\begin{array}{c} A^{*}=\{\langle x,\mu_{A}(x),\nu_{A}(x)\rangle |x\in X\} \end{array}$$ (2)

where $\mu_{A}(x)\to [0,1],\nu_{A}(x)\to [0,1]$ are the belongingness and non-belongingness degrees of an element x in $A^{*}$ with the condition $0⩽\mu_{A}(x)+\nu_{A}(x)⩽1$.

### 2.3. Interval-valued intuitionistic fuzzy set (IVIFS) [33]

An IVIFS $\bar{A}$ over X can be expressed as


$$\begin{array}{c} \bar{A}=\{\langle x,M_{\bar{A}}(x),N_{\bar{A}}(x)\rangle |x\in X\} \end{array}$$ (3)

where $M_{\bar{A}}(x)$ and $N_{\bar{A}}(x)$ ⊂[0,1] are the intervals of members and non-members, respectively, and $\sup M_{\bar{A}}(x)+\sup N_{\bar{A}}(x)\leq 1,\text{ for all }x\in X.$

### 2.4. Construction of IVIFS

Consider the mapping [33] ϕ:IFS→IVIFS described as


$$\begin{array}{c} \phi (A)=\{\langle x,M_{\phi (A)}(x),N_{\phi (A)}(x)\rangle |x\in X\}=\bar{A} \end{array}$$ (4)

where


$$\begin{array}{c} M_{\phi (A)}^{L}(x)=\mu_{A}(x)-k_{1}\cdot \pi_{A}(x),\;0\leq k_{1}\leq \frac{\mu_{A}(x)}{\pi_{A}(x)}, \end{array}$$

$$\begin{array}{c} M_{\phi (A)}^{U}(x)=\mu_{A}(x)+k_{2}\cdot \pi_{A}(x),\;0\leq k_{2}\leq 1, \end{array}$$

$$\begin{array}{c} N_{\phi (A)}^{L}(x)=\nu_{A}(x)-k_{3}\cdot \pi_{A}(x),\;0\leq k_{3}\leq \frac{\nu_{A}(x)}{\pi_{A}(x)}, \end{array}$$

$$\begin{array}{c} N_{\phi (A)}^{U}(x)=\nu_{A}(x)+k_{4}\cdot \pi_{A}(x),\;0\leq k_{4}\leq 1. \end{array}$$

with the conditions:


$$\begin{array}{c} 0\leq k_{2}+k_{4}\leq 1,\;0<k_{2}+k_{1}\leq 1,\;0<k_{4}+k_{3}\leq 1. \end{array}$$

The following is how we define the membership and non-membership widths:


$$\begin{array}{ccc} H_{U} & = & M_{\phi (A)}^{U}(x)-M_{\phi (A)}^{L}(x)=(k_{2}+k_{1})\cdot \pi_{A}(x), \end{array}$$ (5)

$$\begin{array}{ccc} H_{V} & = & N_{\phi (A)}^{U}(x)-N_{\phi (A)}^{L}(x)=(k_{4}+k_{3})\cdot \pi_{A}(x). \end{array}$$ (6)

### 2.5. Pythagorean fuzzy set

Let *X* be an universe of discourse. A set


$$\begin{array}{c} P=\{(x,\xi_{P}(x),\eta_{P}(x))|x\in X\} \end{array}$$ (7)

is called a Pythagorean fuzzy set [25] if it satisfies the condition

$0\leq \xi_{P}^{2}(x)+\eta_{P}^{2}(x)\leq 1$, $\xi_{P}(x),\eta_{P}(x)\in [0,1]$,

where $\xi_{P}(x)$ indicates the membership degree of *x* with regard to *P*, $\eta_{P}(x)$ indicates the non-membership degree of *x* with regard to *P* and

$\zeta_{P}^{2}(x)=1-\xi_{P}^{2}(x)-\eta_{P}^{2}(x)$ is called the hesitation membership degree of *x* with regard to *P*. The pair $\rho =\;(\xi_{P}(x),\eta_{P}(x))$ is referred to as a pythagorean fuzzy number.

### 2.6. Histogram equalization(HE)

A histogram in image processing displays the distribution of gray levels. HE [34] is a technique that redistributes these levels to produce a more uniform distribution, which increases contrast. Nevertheless, the procedure occasionally leads to an excessively bright and contrasted image.

### 2.7. Contrast Limited Adaptive Histogram Equalization (CLAHE)

CLAHE [4] reduces noise by controlling contrast amplification, which enhances AHE. Although CLAHE is generally superior to standard histogram equalization, it can struggle with very dark images. A PFI based strategy is proposed to address this. By specifying the necessary intensity increments before using CLAHE, these images solve the issue of artifacts in dark areas.

### 2.8. Shannon entropy

Shannon entropy [35] calculates the information content of an image. This method uses all available image data to calculate the degree of ambiguity or chance inherent in the image. The following equation expresses the calculation of entropy:


$$\begin{array}{c} SE=-\sum_{i=1}^{l}\sum_{j=1}^{m}\bar{P}(i,j)\log\bar{P}(i,j) \end{array}$$ (8)

where *i* and *j* denote two different intensity values of the images, and $\bar{P}(i,j)$ represents the number of co-occurrences of *i* and *j*.

## 3. Proposed Pythagorean Fuzzy Image (PFI)

To construct the pythagorean fuzzy image (PFI), the source image *I* is first fuzzified using the following normalization process:


$$\begin{array}{c} \mu_{\bar{I}}(\chi {)}_{m\times n}=\frac{\chi_{ij}-\chi_{\min}}{\chi_{\max}-\chi_{\min}}, \end{array}$$ (9)

where $\chi_{ij}$ denotes the luminance value of the (*i*,*j*)th pixel, and $\chi_{\max}$ and $\chi_{\min}$ represent the highest and lowest pixel values of image *I*, respectively.

The membership and non-membership functions are then defined as


$$\begin{array}{c} \bar{N}(\mu_{\bar{I}}(\chi ))=v^{-1}(v(1)-v(\mu_{\bar{I}}(\chi ))) \end{array}$$ (10)

Although parameterized pythagorean fuzzy formulations have been reported previously for tasks such as segmentation and decision-making, those formulations are not designed for pixel-wise contrast redistribution in low-light images. In contrast, the increasing function introduced here is specifically constructed to adaptively regulate membership evolution for low-light image enhancement while satisfying pythagorean fuzzy constraints.

Let us consider the following increasing function:


$$\begin{array}{cc} v(\mu_{\bar{I}}(\chi )) & =\frac{1}{(\alpha +1{)}^{3}}\ln\;(1+(e^{\mu_{\bar{I}}(\chi )}-1)(\alpha +1{)}^{3}),\alpha >0 \end{array}$$ (11)

where


$$\begin{array}{c} \begin{array}{cc} v(0) & =v(\mu_{\bar{I}}(\chi ))=\frac{1}{(\alpha +1{)}^{3}}\ln(1+0)=0 \end{array} \end{array}$$

$$\begin{array}{c} \begin{array}{cc} v(1) & =v(\mu_{\bar{I}}(\chi ))\\ & =\frac{1}{(\alpha +1{)}^{3}}\ln\;(1+(e^{1}-1)(\alpha +1{)}^{3})>0 \end{array} \end{array}$$

with


$$\begin{array}{c} v^{-1}(\mu_{\bar{I}}(\chi ))=\ln\;\frac{e^{\left(\alpha +1{)}^{3}\mu_{\bar{I}}(\chi \right)}+(\alpha +1{)}^{3}-1}{(\alpha +1{)}^{3}} \end{array}$$ (12)

Accordingly,


$$\begin{array}{cc} \bar{N}(\mu_{\bar{I}}(\chi )) & =\ln\;\left(\frac{\left((\alpha +1{)}^{3}-1)e^{\mu_{\bar{I}}(\chi )}+(e-(\alpha +1{)}^{3}+1\right)}{\left(\alpha +1{)}^{3}e^{\mu_{\bar{I}}(\chi )}+(1-(\alpha +1{)}^{3}\right)}\right) \end{array}$$ (13)

where $\bar{N}(1)=0,\bar{N}(0)=1$. Based on the IFS, the degree of membership is computed by applying the following generator:


$$\begin{array}{c} \mu_{\bar{PI}}(\chi )=1-\ln\;\left(\frac{\left((\alpha +1{)}^{3}-1)e^{\mu_{\bar{I}}(\chi )}+(e-(\alpha +1{)}^{3}+1\right)}{\left(\alpha +1{)}^{3}e^{\mu_{\bar{I}}(\chi )}+(1-(\alpha +1{)}^{3}\right)}\right) \end{array}$$ (14)

Using fuzzy negation, the corresponding non-membership degree of the pythagorean fuzzy set is expressed as


$$\begin{array}{c} \nu_{\bar{PI}}(\chi )=\zeta (\mu_{\bar{PI}}(\chi )) \end{array}$$ (15)

$$\begin{array}{c} \nu_{\bar{PI}}(\chi )=\ln\;\left(\frac{\left((\alpha +1{)}^{3}-1)e^{\mu_{\bar{PI}}(\chi )}+(e-(\alpha +1{)}^{3}+1\right)}{\left(\alpha +1{)}^{3}e^{\mu_{\bar{PI}}(\chi )}+(1-(\alpha +1{)}^{3}\right)}\right) \end{array}$$ (16)

Finally, the indeterminacy degree is computed as


$$\begin{array}{c} \pi_{\bar{PI}}(\chi )=\sqrt{1-(\mu_{\bar{PI}}(\chi ){)}^{2}-(\nu_{\bar{PI}}(\chi ){)}^{2}} \end{array}$$ (17)

### 3.1. Defuzzification [36]

Defuzzification is the process of transforming a fuzzy set into a crisp value. In the context of distorted image restoration, an analogous procedure is applied. At the discrete spatial location (*i*,*j*), the fuzzy representation of the pixel’s intensity is mapped to a deterministic, crisp intensity value using a defuzzification function, typically expressed as an equation.


$$\begin{array}{c} \chi_{ij}=\mu_{ij}\cdot (\chi_{\max}-\chi_{\min})+\chi_{\min} \end{array}$$ (18)

Accordingly, the enhanced image is defuzzified at each pixel location (*i*,*j*) using the following equation:


$$\begin{array}{c} DI(i,j)=\frac{EI(i,j)-(k_{1}+k_{2})\cdot \pi_{\bar{PI}}(\chi )}{\mu_{\bar{PI}}} \end{array}$$ (19)

The proposed method introduces a significant advancement over existing fuzzy-based image enhancement techniques, particularly those using IFG and IVIFS. While IFG and IVIFS operate under the linear constraint $\mu_{A}(x)+\nu_{A}(x)⩽1$, the proposed approach employs PFS, which generalizes this relationship to $\xi_{P}^{2}(x)+\eta_{P}^{2}(x)\leq 1$. This quadratic formulation allows for a broader and more flexible representation of uncertainty, especially in low-illumination regions where pixel classification is inherently ambiguous. The hesitation degree $\zeta_{P}^{2}(x)=1-\xi_{P}^{2}(x)-\eta_{P}^{2}(x)$ in PFS provides an additional degree of freedom to adaptively modulate intensity correction during the defuzzification process, thereby enhancing detail preservation in dark areas.

Our method introduces a nonlinear exponential membership generator controlled by a tunable parameter α, which defines a smooth and adaptive mapping of pixel intensities to membership values. While the generator governs the evolution of membership functions, the validity of the pythagorean fuzzy set satisfying the constraint $\xi_{P}^{2}(x)+\eta_{P}^{2}(x)\leq 1$ is ensured through the subsequent construction of the non-membership and hesitation degrees. The combined use of this fuzzy framework with CLAHE enables both global uncertainty modeling and localized contrast enhancement. Together, these innovations lead to improved visual quality and competitive quantitative performance, as demonstrated in the comparative analysis against IFG- and IVIFS-based models.

**Algorithm 1 Pseudo-code of Proposed method for Enhancing Low-Contrast Images**

**Require:** Low-light images $L=[\chi_{ij}{]}_{m\times n}$

1:  **for** *i* = 1 to m **do**

2:   **for** *j* = 1 to n **do**

3:    **Fuzzification:**

$P_{1}=\mu_{\bar{I}}(\chi {)}_{m\times n}=\frac{\chi_{ij}-\chi_{\min}}{\chi_{\max}-\chi_{\min}}$

4:  **end for**

5: **end for**

6:   **for** *i* = 1 to m **do**

7:     **for** *j* = 1 to n **do**

8:      **for** *α* = 0.1:0.1:1.0 **do**

9:         **Compute membership values:**

$\mu_{\bar{PI}}(\chi )=1-\ln\;\left(\frac{\left((\alpha +1{)}^{3}-1)e^{\mu_{\bar{I}}(\chi )}+(e-(\alpha +1{)}^{3}+1\right)}{\left(\alpha +1{)}^{3}e^{\mu_{\bar{I}}(\chi )}+(1-(\alpha +1{)}^{3}\right)}\right)$

10:         **Compute non-membership values:**

$\nu_{\bar{PI}}(\chi )=\ln\;\left(\frac{\left((\alpha +1{)}^{3}-1)e^{\mu_{\bar{PI}}(\chi )}+(e-(\alpha +1{)}^{3}+1\right)}{\left(\alpha +1{)}^{3}e^{\mu_{\bar{PI}}(\chi )}+(1-(\alpha +1{)}^{3}\right)}\right)$

11:         **end for**

12:         **Compute hesitation degree:**

$\pi_{\bar{PI}}(\chi )=\sqrt{1-(\mu_{\bar{PI}}(\chi ){)}^{2}-(\nu_{\bar{PI}}(\chi ){)}^{2}}$

13:  **end for**

14: **end for**

15:  **for** *i* = 1 to m **do**

16:   **for** *j* = 1 to n **do**

17:    **for** *β* = 0.1:0.1:1.0 **do**

18:     **Determine PFIVI image:**

$P=[p_{ij}{]}_{m\times n}=\mu_{\bar{PI}}(\chi )+(k_{1}+k_{2})\cdot \pi_{\bar{PI}}(\chi )$

19:     Apply CLAHE to $P^{\left(\alpha ,\beta \right)}\to P_{c}^{\left(\alpha ,\beta \right)}$

20:    Compute entropy $E^{\left(\alpha ,\beta \right)}$ of $P_{c}^{\left(\alpha ,\beta \right)}$

21:   **end for**

22:  **end for**

23: **end for**

24: **Defuzzification**

$\begin{array}{c} P_{\text{enh}}=\frac{P-(k_{1}+k_{2})\cdot \pi_{\bar{PI}}(\chi )}{\mu_{\bar{PI}}} \end{array}$

**Ensure:** Enhanced image *P*_enh_.

## 4. Experiment and result analysis

The proposed framework was implemented on a Windows 10 (64-bit) system equipped with an Intel(R) Core(TM) i3-1005G1 CPU operating at a base frequency of 1.20 GHz, 4 GB of RAM, and a 1.14 TB hard disk. All experiments were conducted using MATLAB R2023a with the Image Processing Toolbox.

### 4.1. Parameter selection

The proposed enhancement framework employs two key parameters, namely the fuzzification parameter α and the hesitation weighting parameter β, which jointly control the construction of the interval-valued pythagorean fuzzy image. In the experiments, α was uniformly sampled in the range [0.1, 1.0] using 10 discrete values, while β was also varied in the range [0.1, 1.0] with 10 discrete values. This resulted in a total of 100 (α,β) combinations for each input image.

For each parameter pair, the pythagorean fuzzy membership, non-membership, and hesitation degrees were computed using a nonlinear exponential mapping that satisfies the pythagorean fuzzy constraint. The hesitation degree was scaled by the parameter β to regulate the contribution of uncertainty during enhancement. An interval-valued pythagorean fuzzy image was then constructed by combining the membership and weighted hesitation components.

Contrast enhancement was performed by applying contrast-limited adaptive histogram equalization (CLAHE) to the value channel in the HSV color space. Entropy was computed on each CLAHE-enhanced candidate image and used as the selection criterion to identify the optimal output and the corresponding (α,β) pair. All parameter ranges and step sizes were fixed and applied consistently across all datasets to ensure reproducibility.

Following the parameter selection procedure, the enhancement process is carried out according to Algorithm 1. For each (α,β) pair, the input image is fuzzified into membership, non-membership, and hesitation components, and a corresponding interval-valued pythagorean fuzzy image is constructed. CLAHE is then applied to generate a contrast-enhanced candidate image. Entropy is computed for each CLAHE-enhanced candidate, and the (α,β) pair corresponding to the maximum entropy is selected. The final enhanced image is obtained through defuzzification using the selected parameters.

**Data description**: Experiments were conducted on four benchmark low-light datasets: LOL [44], LIME [39], MEF [45], and NPE [46]. The datasets are publicly available from figshare at https://doi.org/10.6084/m9.figshare.27192921. For qualitative evaluation, a sample of 20 images from LOL(1–8), MEF(9–13), LIME(14–17), and NPE(18–20) was shown in Figs 3 and 4. The first column displays the original low light images, and the subsequent columns show results from HE, CLAHE, BPDHE, LightenNet [37], FlightNet [38], LIME [39], IFA [40], NIFG [27], ZeroDCE [41], ZeroDCE++ [42], URetinex [43], and the proposed method.

The results indicate that the proposed method yields competitive and visually improved outputs compared to existing techniques. Entropy values for different methods are compared in Table 1, indicating that the proposed approach achieves higher entropy values, reflecting increased information content. AMBE results are reported in Table 2, where higher values indicate stronger brightness deviation from the corresponding low-light input images rather than improved brightness preservation, and are highlighted accordingly. Quantitative performance in terms of CII is presented in Table 3, where higher values indicate better contrast enhancement and are highlighted. Results for CC and SSIM are reported in Tables 4 and 5, respectively. NIQE results are shown in Table 6, where lower values indicate better perceptual quality and are highlighted accordingly.

**Table 1:** The entropy of the images depicted in Figs 3 and 4.

Item	Original	HE	CLAHE	BPDHE	LightenNet	FLIGHT	LIME	IFA	NIFG	ZeroDCE	ZeroDCE	URetinex	Proposed
					Net [37]	Net [38]	[39]	[40]	[27]	[41]	++ [42]	[43]	
1	5.7638	7.3976	7.0933	5.7206	6.5114	7.3068	6.9388	7.5781	7.7380	7.1828	7.2367	7.4093	7.7543
2	5.7002	7.0050	6.9345	5.6485	6.6725	7.3408	7.1703	7.2774	7.7190	7.2177	7.3696	7.2642	7.7160
3	5.3783	7.2965	6.8939	5.3032	6.3469	7.1274	6.9915	7.4527	7.6123	7.0364	7.1085	7.1326	7.5251
4	6.2095	7.2650	7.2124	6.2121	7.0912	7.2972	7.2589	7.4701	7.6464	7.1494	7.2728	7.4324	7.6438
5	5.2708	7.0067	6.8398	5.2548	6.5749	7.6309	7.0665	7.2176	7.6331	6.9815	7.0732	7.4332	7.7979
6	4.8971	6.8205	6.3337	4.8439	6.1313	7.4141	6.4680	7.1220	7.2398	6.6927	6.7054	6.9848	7.4159
7	5.0047	6.9040	6.3105	4.9179	6.2438	7.3662	6.5301	7.1110	7.2986	6.7184	6.7665	6.9549	7.3492
8	5.2690	7.0714	6.8112	5.2219	6.2641	7.2190	7.0399	7.2399	7.6877	7.0203	7.1592	7.1189	7.6924
9	7.1100	7.4284	7.5134	7.0698	7.8888	7.4948	7.7490	7.6155	7.9352	7.7729	7.8164	7.57	7.8684
10	7.4092	7.3760	7.2686	7.3968	7.4049	7.8622	7.4078	7.6217	7.7838	7.5119	7.5337	7.6797	7.7205
11	4.7738	5.0100	6.1968	3.9938	7.4720	7.4918	7.1151	5.0188	7.4430	6.4472	6.5125	7.3828	7.4625
12	4.8620	6.2108	6.2396	4.7080	7.0961	7.6983	6.7242	6.3905	7.2969	6.5771	6.422	7.3244	7.2898
13	5.8693	6.7629	6.8242	5.8546	7.3106	7.4986	7.1049	7.2114	7.7673	7.1881	7.1839	7.388	7.6557
14	6.8171	7.2585	7.1675	6.7556	6.9729	7.6498	7.1894	7.5204	7.9173	7.6118	7.6992	7.6162	7.8882
15	7.3517	7.3532	7.6275	7.2385	7.7391	7.3432	7.7088	7.5111	7.7993	7.8035	7.7702	6.8416	7.7685
16	4.6172	5.7392	5.4688	4.5287	6.0758	7.0032	5.8649	5.7559	6.4028	5.966	5.9641	6.3815	6.8104
17	5.7314	6.9880	6.8692	5.4635	7.1349	7.4189	7.0152	7.1190	7.8088	7.1996	7.3201	7.5895	7.7475
18	7.5966	7.6628	7.7392	7.5486	7.6866	7.5000	7.8208	7.6900	7.8719	7.8301	7.8217	7.3264	7.8306
19	7.2118	7.3863	7.2533	7.1867	7.3978	7.8628	7.1643	7.6422	7.7754	7.4392	7.5495	7.6927	7.7382
20	7.0289	7.1327	7.7720	6.8913	7.7186	7.1223	7.8956	7.4980	7.8777	7.849	7.8829	6.7725	7.85

**Table 2:** The AMBE of the images depicted in Figs 3 and 4.

Item	HE	CLAHE	BPDHE	Lighten	Flight	LIME	IFA	NIFG	ZeroDCE	ZeroDCE	URetinex	Proposed
				Net [37]	Net [38]	[39]	[40]	[27]	[41]	++ [42]	[43]	
1	80.1480	38.6989	2.4270	23.0736	68.7676	60.8358	95.5675	106.3709	67.2394	74.9866	103.4485	**121.0172**
2	90.3166	31.1463	2.8181	27.2955	85.8030	58.8939	101.2610	102.3831	58.177	64.3115	100.7101	**121.0608**
3	94.3431	37.8076	2.5851	19.2222	71.6121	61.4536	106.7361	111.2017	62.6056	69.2166	102.4559	**126.8092**
4	78.7913	28.2274	2.4605	28.7780	57.5802	61.1285	90.1157	90.7049	66.1685	68.3042	94.0551	**98.2659**
5	92.7300	33.1713	2.8412	22.4889	93.9448	52.9965	108.1196	107.4905	55.7075	58.0628	99.0042	**126.3487**
6	92.8841	23.9687	2.7175	21.4815	123.3692	54.6553	108.2368	105.2545	63.5733	61.9511	102.3319	**152.6416**
7	99.6535	24.6129	2.6706	23.1424	126.4456	59.2501	109.8382	110.2808	66.039	64.6613	106.3997	**159.7669**
8	93.8252	34.7765	2.7987	17.6037	81.1541	57.7574	104.3837	111.8469	56.5782	63.408	100.9685	**126.1199**
9	56.5306	17.6170	2.5797	79.1369	**108.8821**	57.0145	60.6416	72.8499	54.9961	61.0398	70.8928	72.6577
10	22.7646	4.7387	2.6418	69.6401	**73.8496**	42.7352	46.9624	63.1933	55.8565	57.1472	58.8269	72.7847
11	105.3083	23.107	3.3484	78.7032	76.1320	47.8703	**106.0147**	74.5207	36.3468	41.0617	83.7645	100.8778
12	98.6617	20.9247	3.2779	48.6425	102.8412	45.6229	103.5138	84.8047	41.7996	44.8004	87.357	**107.0955**
13	47.3753	12.3355	2.8525	39.2110	**101.7991**	38.5470	76.4573	93.6626	46.963	49.1203	71.26	74.3484
14	34.1968	23.7606	2.8456	22.4543	42.9721	39.1086	64.4607	**81.4556**	55.5513	62.1379	63.5125	80.1272
15	26.6437	19.7254	2.7235	37.8380	50.6172	43.3541	29.7442	54.2570	41.1728	48.9948	52.2367	**58.6835**
16	67.1047	8.3575	3.4649	20.3900	**94.3663**	34.7065	69.2467	71.6684	34.5202	35.0769	62.1342	64.5178
17	77.9854	29.1943	2.9030	47.5650	58.7801	49.0600	104.9750	100.8825	53.0211	56.5576	85.2162	**104.9060**
18	27.0458	20.0158	2.6213	46.7459	**71.5088**	49.3212	33.7641	56.7040	47.1503	54.275	56.5459	59.1028
19	30.3078	17.3342	2.4851	48.9568	56.7349	47.8359	57.5801	69.3743	65.6076	69.4025	66.9574	**81.8567**
20	49.1289	30.2849	2.6628	38.1300	64.3646	53.0288	52.7980	62.3675	45.5156	54.3903	60.9789	**65.6293**

**Table 3:** The CII of the images depicted in Figs 3 and 4.

Item	HE	CLAHE	BPDHE	Lighten	Flight	LIME	IFA	NIFG	ZeroDCE	ZeroDCE	URetinex	Proposed
				Net [37]	Net [38]	[39]	[40]	[27]	[41]	++ [42]	[43]	
1	4.2825	2.5849	1.0994	1.9450	3.8164	3.4923	4.9140	5.3565	3.7538	4.0711	5.2368	**5.9563**
2	5.2766	2.4748	1.1334	2.2925	5.0628	3.7887	5.7948	5.8479	3.7547	4.0452	5.7687	**6.7323**
3	6.0458	3.0221	1.1383	2.0281	4.8300	4.2867	6.7086	6.9474	4.3483	4.7019	6.4797	**7.7822**
4	3.3650	1.8473	1.0739	1.8638	2.7283	2.8348	3.7409	3.7226	2.9861	3.0502	3.8231	**3.9495**
5	7.1711	3.2075	1.1891	2.4966	7.2520	4.5269	8.1953	8.1534	4.7073	4.864	7.5887	**9.4084**
6	7.3756	2.6452	1.1865	2.4745	9.4681	4.7515	8.4294	8.2247	5.3637	5.2523	8.0241	**11.4773**
7	7.3739	2.5742	1.1708	2.4802	9.0875	4.7896	8.0253	8.0536	5.2239	5.1357	7.8053	**11.2187**
8	6.6535	3.0955	1.1686	2.0607	5.8900	4.4802	7.2897	7.7394	4.4091	4.8207	7.0839	**8.5994**
9	1.984	1.3068	1.0449	2.3783	**2.8964**	1.9930	2.0562	2.2687	1.9579	2.0631	2.2347	2.2655
10	1.3311	1.0689	1.0384	2.0129	**2.0741**	1.6216	1.6830	1.9191	1.8124	1.8312	1.8556	2.0586
11	8.1525	2.5717	1.2274	6.3455	6.1709	4.2513	**8.2005**	6.0614	3.4687	3.7889	6.6893	7.8516
12	9.8816	2.8837	1.2951	5.3788	10.2578	5.1070	10.3184	8.6342	4.7628	5.0329	8.8639	**10.6408**
13	2.3156	1.3426	1.0792	2.0889	**3.8269**	2.0704	3.1232	3.6010	2.3041	2.364	2.9789	3.0646
14	1.8917	1.6196	1.0742	1.5855	2.1205	2.0198	2.6808	**3.1240**	2.4485	2.6203	2.6561	3.0893
15	1.3399	1.2516	1.0347	1.4827	1.6457	1.5531	1.3795	1.6922	1.5252	1.625	1.6664	**1.7486**
16	3.4175	1.3011	1.1248	1.7346	**4.3996**	2.2503	3.4946	3.5819	2.2436	2.2637	3.2384	3.3243
17	5.2551	2.5929	1.1584	3.5953	4.2072	3.6769	**6.7278**	6.5045	3.893	4.086	5.6497	6.7240
18	1.3272	1.2421	1.0317	1.5655	**1.8651**	1.5967	1.4085	1.6860	1.5704	1.6566	1.6841	1.7150
19	1.5572	1.3187	1.0457	1.8996	2.0430	1.8794	2.0586	2.2748	2.2061	2.2759	2.231	**2.5049**
20	1.7082	1.4365	1.0384	1.5497	1.9279	1.7644	1.7610	1.8991	1.6561	1.7841	1.8791	**1.9461**

**Table 4:** The Correlation coefficient of the images depicted in Figs 3 and 4.

Item	HE	CLAHE	BPDHE	Lighten	Flight	LIME	IFA	NIFG	ZeroDCE	ZeroDCE	URetinex	Proposed
				Net [37]	Net [38]	[39]	[40]	[27]	[41]	++ [42]	[43]	
1	0.9588	0.9474	0.9975	0.9748	0.9912	0.9230	0.9517	0.9528	0.9389	0.9411	0.9654	0.9569
2	0.9318	0.9518	0.9984	0.9644	0.9863	0.9084	0.9333	0.9368	0.948	0.9423	0.9691	0.9552
3	0.9462	0.9550	0.9961	0.9415	0.9881	0.8926	0.9348	0.9276	0.9263	0.9214	0.9627	0.9269
4	0.8393	0.9103	0.9995	0.9434	0.9849	0.8900	0.8583	0.8433	0.8188	0.859	0.8796	0.8401
5	0.9739	0.9799	0.9962	0.9202	0.9732	0.9429	0.9514	0.9438	0.9446	0.9566	0.9605	0.9330
6	0.9830	0.8918	0.9919	0.9908	0.9715	0.9442	0.9502	0.9730	0.9759	0.9731	0.9672	0.9448
7	0.9817	0.8971	0.9932	0.9857	0.9672	0.9221	0.9692	0.9541	0.9517	0.9569	0.9661	0.9199
8	0.9862	0.9947	0.9961	0.9641	0.9896	0.9186	0.9745	0.9748	0.977	0.9631	0.9878	0.9748
9	0.9205	0.8576	0.9999	0.7933	0.8153	0.9300	0.9136	0.8546	0.9072	0.8982	0.9371	0.8535
10	0.9910	0.8546	0.9998	0.7631	0.9546	0.9319	0.9892	0.9203	0.8907	0.8974	0.9743	0.9093
11	0.7217	0.9499	0.9989	0.3922	0.8957	0.8889	0.7272	0.8769	0.9145	0.906	0.8949	0.8836
12	0.8085	0.9736	0.9973	0.9286	0.8709	0.8759	0.8185	0.8547	0.9145	0.9195	0.8758	0.834
13	0.8956	0.9659	0.9998	0.8815	0.8058	0.9689	0.8235	0.8075	0.9234	0.9097	0.9192	0.8847
14	0.8801	0.7747	0.9994	0.8702	0.9836	0.8284	0.9585	0.9116	0.8796	0.8768	0.9529	0.9188
15	0.9249	0.9042	0.9999	0.9533	0.9740	0.9069	0.9325	0.9311	0.9297	0.9221	0.9805	0.9370
16	0.6244	0.9815	0.9999	0.9875	0.7345	0.9649	0.6368	0.8479	0.9401	0.9354	0.9363	0.9060
17	0.7252	0.8523	0.9987	0.7248	0.9156	0.7850	0.7975	0.8029	0.8569	0.8515	0.8223	0.8169
18	0.9836	0.9026	0.9999	0.9590	0.9327	0.9407	0.9821	0.9222	0.9291	0.9243	0.9781	0.9189
19	0.9723	0.8083	0.9997	0.6302	0.9678	0.8398	0.9571	0.9036	0.8423	0.8725	0.9561	0.8968
20	0.8757	0.8239	0.9999	0.9542	0.9376	0.9036	0.8878	0.8714	0.9187	0.8996	0.9588	0.8656

**Table 5:** The SSIM of the images depicted in Figs 3 and 4.

Item	HE	CLAHE	BPDHE	Lighten	Flight	LIME	IFA	NIFG	ZeroDCE	ZeroDCE	URetinex	Proposed
				Net [37]	Net [38]	[39]	[40]	[27]	[41]	++ [42]	[43]	
1	0.2313	0.4790	0.9818	0.7327	0.3086	0.3371	0.1828	0.1873	0.3513	0.3148	0.2619	0.2170
2	0.1770	0.4935	0.9552	0.6310	0.2453	0.2615	0.1598	0.1611	0.3043	0.2743	0.218	0.1804
3	0.1235	0.3860	0.9710	0.6918	0.2457	0.2321	0.1070	0.1245	0.2654	0.236	0.1916	0.1530
4	0.3102	0.5891	0.9592	0.6774	0.4827	0.3844	0.2797	0.2915	0.4117	0.4009	0.3477	0.3425
5	0.1290	0.4327	0.9264	0.6028	0.1515	0.2308	0.0917	0.1094	0.2539	0.239	0.1694	0.1264
6	0.1636	0.5583	0.9365	0.6450	0.1179	0.2725	0.1122	0.1315	0.26	0.2652	0.1965	0.1270
7	0.1335	0.5460	0.9526	0.6356	0.1398	0.2592	0.1003	0.1293	0.2633	0.2666	0.2004	0.1353
8	0.1083	0.4026	0.9599	0.6880	0.1910	0.2122	0.0918	0.1047	0.2513	0.2192	0.1697	0.1239
9	0.5237	0.6290	0.9658	0.4116	0.2884	0.4864	0.5045	0.4318	0.5167	0.4887	0.4494	0.4404
10	0.8215	0.6835	0.9587	0.5823	0.5577	0.6616	0.7258	0.5754	0.6417	0.6344	0.6571	0.5515
11	0.1000	0.4544	0.8153	0.1982	0.1335	0.1736	0.1013	0.1259	0.2773	0.2476	0.147	0.1226
12	0.0798	0.4613	0.8402	0.2502	0.0873	0.2237	0.0797	0.1064	0.2818	0.264	0.1703	0.0887
13	0.4456	0.6915	0.9277	0.4627	0.1829	0.4458	0.3251	0.2529	0.4291	0.4164	0.3414	0.3247
14	0.6332	0.6257	0.9501	0.6536	0.6265	0.5107	0.4829	0.3489	0.4657	0.4374	0.4532	0.4098
15	0.6199	0.6653	0.9658	0.6498	0.6551	0.5534	0.6088	0.5526	0.5979	0.5688	0.6007	0.5688
16	0.1957	0.6882	0.8655	0.4042	0.1784	0.3119	0.1958	0.1859	0.3303	0.3246	0.2423	0.2238
17	0.1736	0.4254	0.9159	0.2742	0.3050	0.2524	0.1373	0.1310	0.2692	0.2499	0.1966	0.1731
18	0.8194	0.7247	0.9819	0.7107	0.6239	0.6365	0.7916	0.6196	0.6517	0.6282	0.6673	0.6088
19	0.8224	0.7670	0.9776	0.6251	0.6787	0.6396	0.7011	0.5512	0.5909	0.5765	0.604	0.5095
20	0.4895	0.4986	0.9717	0.6062	0.4577	0.4654	0.4809	0.4461	0.5173	0.4793	0.473	0.4385

**Table 6:** The NIQE of the images depicted in Figs 3 and 4.

Item	HE	CLAHE	BPDHE	Lighten	Flight	LIME	IFA	NIFG	ZeroDCE	ZeroDCE	URetinex	Proposed
				Net [37]	Net [38]	[39]	[40]	[27]	[41]	++ [42]	[43]	
1	6.8489	5.6894	4.0695	4.4919	**3.7351**	6.3126	6.8993	5.8833	5.579	5.999	3.8988	3.865
2	8.3078	8.0926	5.776	6.8297	**3.1513**	7.8669	7.008	7.2889	6.4818	6.4933	4.0294	4.351
3	6.5234	5.6622	4.4096	4.9889	3.8926	4.1424	6.4582	6.2847	5.931	6.2395	3.8688	**3.4819**
4	6.2939	6.62	5.7019	6.0802	**3.0231**	3.8135	5.9869	6.7794	5.7487	5.8897	4.1889	3.6639
5	8.1125	7.5601	5.6519	7.0538	**3.8312**	8.8193	7.8655	7.7119	7.2756	7.7586	4.0378	5.2143
6	9.2366	8.1576	6.9163	7.6956	**2.813**	9.1692	9.734	8.7898	8.2647	8.5278	2.8638	5.2124
7	9.2628	7.6215	6.3921	7.5136	**3.4486**	9.8241	9.388	8.4537	8.538	8.7483	3.593	4.5855
8	9.1738	8.9276	6.9562	7.8172	**5.1676**	9.875	8.8847	8.9759	8.3023	8.1897	5.8937	5.6775
9	2.1676	2.0316	2.4592	2.1823	2.9624	2.1727	2.1475	1.9685	2.0377	2.0468	2.6843	**1.9256**
10	3.3083	2.8103	3.6967	3.2404	3.4757	3.465	3.3938	2.9174	3.0228	3.1815	**2.7186**	2.8984
11	5.073	3.6612	4.9905	4.2531	3.4982	5.741	5.0356	4.3918	3.9698	3.9872	3.3636	**3.0709**
12	3.0446	2.9701	4.0981	3.0103	**2.9663**	4.1454	3.0271	3.5268	3.4571	3.4638	3.2106	3.4201
13	6.5305	4.9017	6.3087	5.4193	4.3064	5.655	5.8663	5.5925	5.5056	5.5799	4.2428	**4.1787**
14	2.2231	2.4835	2.5924	**2.1245**	2.1388	2.5605	2.5832	2.4179	2.3289	2.4054	2.9316	2.1558
15	5.4135	4.7546	5.615	4.7795	**4.4097**	4.6947	5.5594	6.0282	4.4716	4.8753	5.1833	4.8433
16	4.1595	4.0539	5.7929	4.1186	**3.3584**	5.1577	4.3281	5.0755	4.8168	4.8347	4.1896	3.5901
17	2.8051	2.8403	3.8088	2.6814	2.4035	3.3828	2.3327	2.8495	2.9337	3.0086	2.6129	**2.1262**
18	3.1598	3.6623	3.0481	2.7884	**2.7376**	3.3438	3.2455	3.6014	3.1489	3.286	3.4268	3.1157
19	3.3794	3.5553	3.2406	3.2147	3.9664	3.6905	3.5737	3.234	3.7948	3.8451	3.3696	**3.0846**
20	4.4739	4.5713	4.5167	4.4115	4.7084	4.6004	4.3241	4.5455	4.3717	3.9967	5.8266	**3.9373**

**Fig 1:**
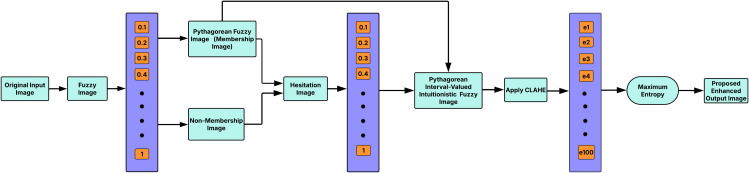
Methodology of the proposed method.

**Fig 2:**
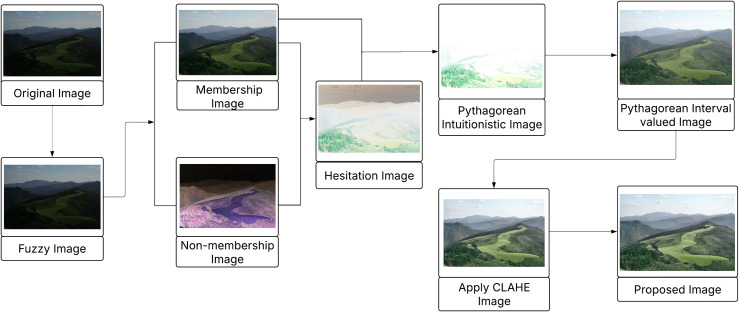
Image transformation of the proposed method.

**Fig 3:**
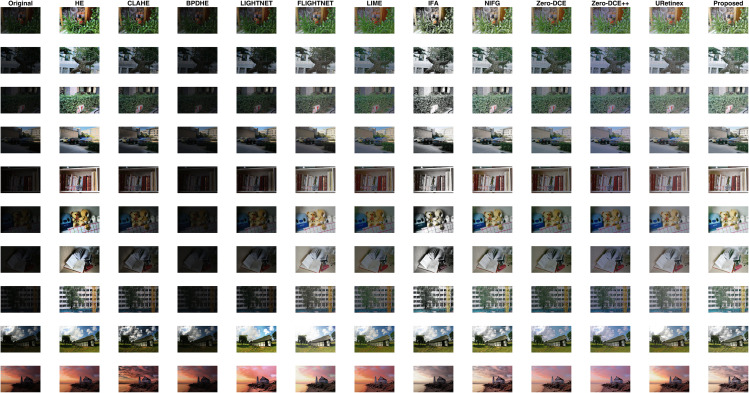
Comparison of image enhancement results from different techniques.

**Fig 4:**
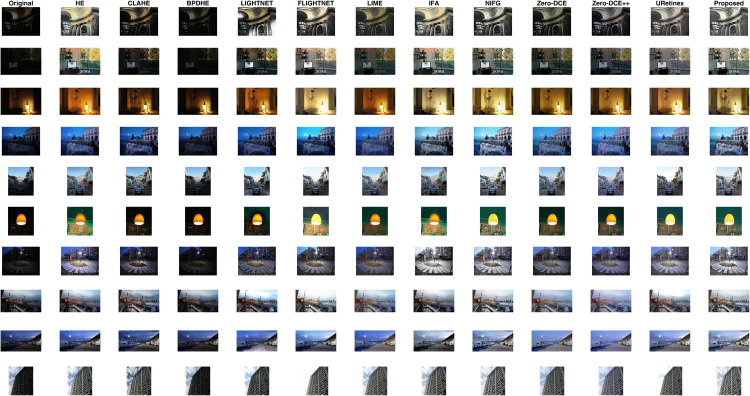
Comparison of image enhancement results from different techniques.

The fuzzy-based enhancement process is summarized in Algorithm 1, the workflow of the proposed technique is presented in Fig 1 and visualized using an image in Fig 2, and the comparative analysis of competing methods is presented in Figs 3 and 4. To preserve color fidelity in low light conditions, the hesitation degree regulates pixel-level enhancement intensity. This mechanism prevents over-enhancement in uniform or chromatically sensitive regions, thereby maintaining natural color tones. Additionally, applying CLAHE only after the fuzzy transformation helps avoid early noise amplification. Although the proposed method does not explicitly include a noise suppression stage, the uncertainty modeling through hesitation inherently reduces the enhancement weights for noisy or ambiguous pixels, leading to a more visually balanced result.

### 4.2. Performance metrics

#### 4.2.1. Absolute Mean Brightness Error (AMBE).

AMBE [47] measures the absolute difference between the mean brightness values of the original and enhanced images and is commonly used to assess brightness preservation during image enhancement. Formally, lower AMBE values indicate better preservation of the original image brightness, whereas higher AMBE values correspond to larger brightness deviations introduced by the enhancement process.


$$\begin{array}{c} AMBE(OI,EO)=|mb(OI)-mb(EO)| \end{array}$$ (20)

where *mb*(*OI*) and *mb*(*EO*) denote the mean brightness of the original input image and the enhanced output image, respectively. In the context of low-light image enhancement, strict brightness preservation is not always desirable, as effective illumination correction typically requires an increase in overall brightness. Therefore, in this work, AMBE is reported in Table 2 to quantify the degree of brightness modification introduced by the enhancement process rather than as a standalone indicator of visual quality.

#### 4.2.2. Contrast improvement index (CII).

CII [48] measures the change in contrast between an original and enhanced output image. A CII value greater than 1 indicates improved contrast in the enhanced image.

The Contrast Improvement Index (CII) is defined as:


$$\begin{array}{c} CII=\frac{C_{EO}}{C_{OI}} \end{array}$$ (21)

where OI represents the mean intensity value of the original image and EO refers to the mean intensity value of the corresponding enhanced image. The proposed image enhancement method shows superior performance compared to existing methods, as demonstrated by the comparative results presented in Table 3.

#### 4.2.3. Correlation coefficient (CC).


$$\begin{array}{c} CC=\frac{\sum_{i=1}^{n}(x_{i}-\bar{x})(y_{i}-\bar{y})}{\sqrt{\sum_{i=1}^{n}(x_{i}-\bar{x}{)}^{2}\sum_{i=1}^{n}(y_{i}-\bar{y}{)}^{2}}} \end{array}$$ (22)

where $x_{i},y_{i}$ denotes the mean gray values of the corresponding pixels in the enhanced picture and the high resolution images and $\bar{x},\bar{y}$ denotes the average gray value of both images [27]. If the correlation coefficient is closer to 1, it indicates that there is a minimal change between the original and enhanced images, where the information of the image is largely preserved. If the correlation coefficient is close to 0, it indicates that there is a significant difference between them, which implies that the enhancement would have destroyed the original details. In our work, it is shown that the correlation coefficient values are neither maximum nor minimum as in Table 4. This shows that the proposed method enhances the original image by preserving the details but not destroying it, as the values are closer to 1.

#### 4.2.4. Structural Similarity Index (SSIM).


$$\begin{array}{c} \text{SSIM}(i,o)=\frac{\left(2{\bar{\mu }}_{i}{\bar{\mu }}_{o}+c_{1})(2\sigma_{io}+c_{2}\right)}{\left({\bar{\mu }}_{i}^{2}+{\bar{\mu }}_{o}^{2}+c_{1})(\sigma_{i}^{2}+\sigma_{o}^{2}+c_{2}\right)} \end{array}$$ (23)

where *c*_1_ and *c*_2_ are positive constants. i and o represent original and enhanced images. ${\bar{\mu }}_{i}$ and ${\bar{\mu }}_{o}$ represent the mean intensities of i and o respectively. $\sigma_{io}$ represents the covariance of i and o. $\sigma_{i}^{2}$ and $\sigma_{o}^{2}$ are the variances of i and o, respectively. The SSIM [49] values of the proposed method are displayed in Table 5.

## 5. Ablation study

**Fig 5:**
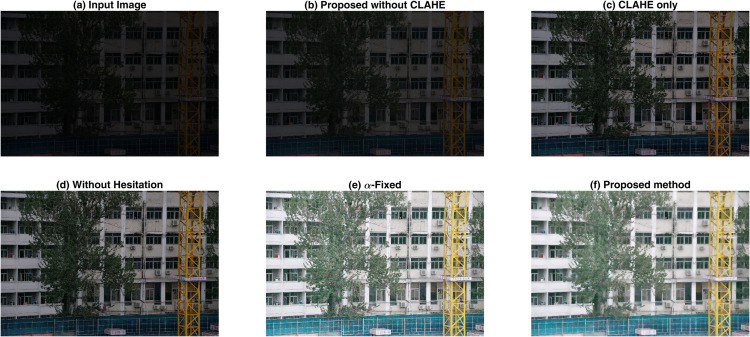
Visual comparison of ablation study variants.

An ablation study on 5 representative images from the LOL(600x400 resolution) and MEF datasets evaluated five configurations: PFS with fixed α, PFS without hesitation, PFS only, CLAHE only, and the proposed method. The observed average values of entropy, AMBE, CII, CC, and SSIM are presented in Table 7, and the visual representation is shown in Fig 5 of a single image. The results show that fixing α or removing hesitation reduces adaptability; PFS alone preserves structure but lacks contrast enhancement, while CLAHE alone boosts contrast but may cause brightness inconsistencies. The proposed method achieves a balanced improvement by combining the strengths of both approaches.

**Table 7:** Ablation study on representative images from the LOL and MEF datasets.

Method	Entropy(Avg.)	AMBE (Avg.)	CII (Avg.)	CC (Avg.)	SSIM (Avg.)
PFS Only	5.4025	51.0512	3.9091	0.9695	0.4794
CLAHE Only	6.7724	28.9086	2.5798	0.9673	0.4705
Without Hesitation	7.2626	54.3671	3.91754	0.9578	0.2846
α-Fixed	7.5676	114.346	7.3996	0.9159	0.1517
PFS + CLAHE (Ours)	7.5977	111.0787	7.2087	0.9151	0.1528

## 6. Time complexity analysis

The computational efficiency of the proposed method was evaluated against LIME, IFA, NIFG, and LightNet using the 20 test images presented in Figs 3 and 4. The execution times (in seconds) for each method are summarized in Table 8. The results demonstrate that the proposed method achieves a balanced trade off between processing speed and image quality, making it suitable for near real-time low light enhancement tasks, particularly in resource constrained environments.

**Table 8:** Time complexity of the images shown in Figs 3 and 4.

Images	LIME	IFA	NIFG	LightNet	Proposed
1	4.20	20.02	6.66	13.56	9.19
2	1.36	20.05	6.58	17.58	10.90
3	0.95	19.15	6.69	16.18	9.86
4	0.86	18.77	6.22	14.76	9.70
5	0.93	18.25	6.57	12.99	8.66
6	0.86	18.32	6.41	13.03	8.57
7	0.92	19.29	6.44	14.12	10.05
8	0.78	18.87	6.69	14.20	9.71
9	0.69	14.64	4.98	14.61	5.73
10	1.00	14.33	4.76	13.28	6.75
11	0.71	15.19	4.91	12.69	5.89
12	0.77	12.46	4.85	11.99	10.3
13	0.69	14.93	5.40	12.34	7.90
14	11.62	18.20	0.87	11.56	8.33
15	1.91	12.33	0.61	15.38	5.72
16	1.48	16.80	0.79	17.35	9.94
17	1.61	59.27	2.61	29.29	24.5
18	1.62	18.71	0.77	16.21	8.72
19	1.10	14.94	0.65	14.06	6.45
20	0.99	14.28	0.73	14.26	7.35

## 7. Advantages and limitations

The benefits of the suggested approach are as follows:

a) The introduced pythagorean fuzzy method effectively tackles the issues related to the enhancement of low light images.
b) Comparative evaluation, illustrated in Table 1, shows that the proposed algorithm significantly enhances image quality compared to current techniques.
c) The suggested method delivers benchmark results that exceed those of previous approaches, as outlined in the performance metrics (Tables 1-6).
d) The combination of the pythagorean fuzzy method with CLAHE produces better outcomes when compared with other existing techniques.

**Limitations**:

a) The proposed method is specifically optimized for enhancing low light images.
b) Although it effectively preserves image details, it may introduce noise in very dark regions of low contrast images during the enhancement process.

## 8. Conclusion

This study proposes a novel pythagorean intuitionistic fuzzy enhancement technique for low light images. By adapting the CLAHE framework through the proposed method, improved image quality is achieved. Experimental results demonstrate that the technique effectively enhances color and contrast while preserving the natural appearance of the images. Compared to conventional methods such as HE, CLAHE, BPDHE, LightenNet, FlightNet, LIME, IFA, NIFG, ZeroDCE, ZeroDCE++, URetinex, and the proposed approach yields superior performance. Quantitative evaluation using metrics such as entropy, AMBE, CII, CC, SSIM, and NIQE confirms the method’s effectiveness in enhancing both the visual quality and structural integrity of images. Future work may extend this technique to various datasets and explore its application in video enhancement.

## Supporting information

S1 File Images presented in Figs 3 and 5. (RAR)

S2 File Images presented in Fig 4. (RAR)
